# Role of lncRNA FAM83H antisense RNA1 (FAM83H-AS1) in the progression of non-small cell lung cancer by regulating the miR-545-3p/heparan sulfate 6-O-sulfotransferase (HS6ST2) axis

**DOI:** 10.1080/21655979.2022.2031668

**Published:** 2022-03-09

**Authors:** Yue Zhang, Yue Yu, Xuchen Cao, Peng Chen

**Affiliations:** a Department of Thoracic Oncology, Lung Cancer Diagnosis and Treatment Center, Tianjin Medical University Cancer Institute and Hospital, National Clinical Research Center for Cancer, Key Laboratory of Cancer Prevention and Therapy, Tianjin, Tianjin’s Clinical Research Center for Cancer, Tianjin, China; b Department of Mammography Surgery, The First Affiliated Hospital of HeBei North University, Zhangjiakou, Hebei, China; c The First Surgical Department of Breast Cancer, Tianjin Medical University Cancer Institute and Hospital, National Clinical Research Center for Cancer, Key Laboratory of Cancer Prevention and Therapy, Tianjin, Tianjin’s Clinical Research Center for Cancer, Tianjin, China

**Keywords:** Lung cancer, NSCLC, FAM83H-AS1, miR-545-3p, HS6ST2

## Abstract

Long non-coding RNAs (lncRNAs) are crucial regulators of cancer pathogenesis and are potentially useful diagnostic and prognostic biomarker tools. FAM83H antisense RNA1 (FAM83H-AS1) has been reported to be a vital regulator of different cancers; however, little attention has been paid to its significance in lung cancer. Non-tumorigenic lung cell line BEAS-2B and adenocarcinoma lung cancer cell lines NCI-H1299 and HCC827 were used in the present study. In addition, RNA immunoprecipitation, Western blotting, quantitative reverse transcription-PCR (qRT-PCR), and luciferase reporter assays were used to dissect the role of FAM83H-AS1 in lung cancer progression. The results revealed that FAM83H-AS1 is highly expressed in lung cancer tissues, and its knockdown inhibits lung cancer cell invasion and proliferation reducing tumor growth *in vivo*. Besides, we found that FAM83H-AS1 targets miR-545-3p, and a negative correlation exists between their expression in lung cancer tissues. Simultaneously, miR-545-3p negatively regulates heparan sulfate 6-O-sulfotransferase (HS6ST2). Moreover, inhibition of miR-545-3p promoted HS6ST2 protein expression and lung cancer cell invasion. FAM83H-AS1 favors non-small cell lung cancer by targeting the miR-545-3p/HS6ST2 axis, supporting the possibility of developing FAM83H-AS1 as a target for NSCLC intervention.

## Graphical abstract

**Figure:**
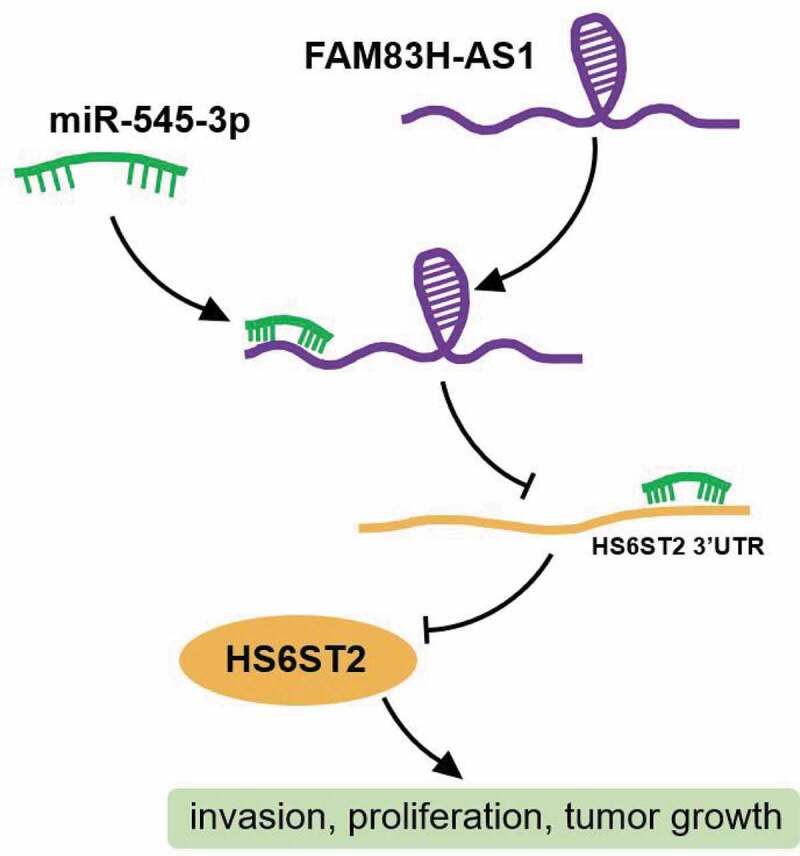

## Introduction

Histopathologically, lung cancer is classified as small cell lung cancer (SCLC) or non-small cell lung cancer (NSCLC), where NSCLC is the predominant subtype and a leading cause of cancer-associated deaths globally [1,2]. NSCLC is characterized by heterogeneity, with various subtypes representing different clinical outcomes and indices that require specific treatment strategies [3–5]. Undeniably, complex cellular signaling and tumor microenvironment factors associated with poor prognosis impart a distinct bio-pathological condition to each disorder [1]. The identification of oncogenic driver modulation alleviated the effects of lung cancer; however, most patients have no actionable molecular abnormalities [6,7]. Therefore, the identification of new biomarkers and alternative treatments is necessary.

Long non-coding RNAs (lncRNAs) are RNA transcripts larger than 200 bp and show specific patterns in healthy and tumor tissues [8–11]. Although most lncRNAs are yet to be discovered, they have emerged as novel cancer mediators [12–17] involved in almost all tumor cell malignant behaviors [13,17–20]. For example, lncRNA DANCR is abundantly expressed in lung tissues and is linked to the advanced tumor progression [21]. Similarly, lncRNA TUC338 in lung cancer is associated with activation of the MAPK pathway [22], whereas lncRNA MCM3AP accelerates the malignant progression of SCLC cells [23]. FAM83H antisense RNA1 (FAM83H-AS1) is a potential modulator of oncogenes in various tumors [24]. Furthermore, enhanced expression of FAM83H-AS1 is associated with the malignant phenotypes of lung cancer *in vitro* and *in vivo* [25]. However, the underlying mechanisms have not been completely elucidated.

MicroRNAs (miRNAs) are endogenous, small non-coding RNAs that regulate gene expression. Riveting evidence has shown that miRNA expression is distinctly dysregulated in human cancer, including the deletion or amplification of miRNA genes, dysregulated epigenetic changes, unusual miRNA transcriptional control, and defective miRNA biogenesis mechanism [26]. Studies have shown that miR-545-3p acts as a tumor suppressor and post-transcriptional regulator of various genes in lung cancer [27,28].

Heparan sulfate 6-O-sulfotransferase (HS6ST) is involved in various biological processes [29,30]. Specifically, HS6ST2 is involved in the pathogenesis of malignant tumors and up regulated in different tumor types, such as thyroid [31,32], colorectal [33], and lung cancers [33,34].

The role of FAM83H-AS1 in lung cancer and its underlying mechanism require detailed analysis. Additionally, understanding the significance of miR-545-3p and HS6ST2 in lung cancer pathogenesis would provide a valuable theoretical basis for the diagnosis and treatment of NSCLC. In present study, the FAM83H-AS1/miR-545-3p/HS6ST2 axis was assumed a novel signaling pathway associated with lung cancer progression and its in-depth molecular mechanism was investigated.

## Materials and methods

### Clinical samples

A total of 32 patients diagnosed with NSCLC at Tianjin Medical University Cancer Institute and Hospital (Tianjin, China) were selected for sample collection. Cancerous and normal lung tissue specimens from each patient. Tissue specimens were collected after surgical resection and promptly transferred to liquid nitrogen for future use. A written informed consent was obtained from all the patients. This study was approved by the ethical committee of the Tianjin Medical University Cancer Institute and Hospital (Tianjin, China). The baseline characteristics of the patients are listed in Supplementary Table 1.

### Cell lines and cell culture

The human non-tumorigenic lung epithelial cell line BEAS-2B (ATCC, USA) was cultured in BEBM complete medium supplemented with 10% fetal bovine serum (FBS), penicillin (100 U/ml, MD, USA), streptomycin (100 mg/ml, MD, USA), and incubated at 37°C in 5% CO_2_ atmosphere. The adenocarcinoma lung cancer cell lines NCI-H1299, NCI-H1650, and HCC827 (ATCC, USA) were cultured in RPMI-1640 medium supplemented with 10% FBS, penicillin (100 U/ml), streptomycin (100 mg/ml), and incubated at 37°C in 5% CO_2_.

### Cell transfection

The siRNAs targeting FAM83H-AS1 (si-FAM83H-AS1), HS6ST2 (si-HS6ST2), siRNA negative control (si-NC), miR-545-3p mimic or mimic NC, and miR-545-3p inhibitor or inhibitor NC were provided by GenePharma, China. HCC827 and NCI-H1650 cells were transfected using the X-tremeGENE transfection reagent (Thermo Fisher Scientific, USA) according to the manufacturer’s protocol. After 48 h of transfection, quantitative reverse transcription-PCR (qRT-PCR) was performed to test the transfection efficiency. The sequences of the plasmids used in this assay are listed in Supplementary Table 2.

### qRT-PCR

Total RNA from cultured cells was extracted using TRIzol reagent. cDNA was synthesized using a cDNA synthesis kit (Roche, USA) or one-step miRNA RT kit (Haigene, China), according to the manufacturer’s protocol. The miR-545-3p, FAM83H-AS1 (GenBank NM_001381875), and HS6ST2 mRNA levels were quantified by qRT-PCR using Real-Time PCR Master Mix (Genema, China). The relative expression levels of miR-545-3p, FAM83H-AS1, and HS6ST2 were calculated using the 2^−∆∆^Cq method [35], with normalization to U6 and GAPDH. The primers used are listed in Table 1.

**Table 1.** The sequences of the primers in this study

Primer	Sequences
FAM83S-AS1	Forward: 5′-TAGGAAACGAGCGAGCCC-3′
	Reverse: 5′-GCTTTGGGTCTCCCCTTCTT-3′
HS6ST2	Forward: 5′-GAAGGCAGAACTCAGGCAAGG-3′
	Reverse: 5′-CCAATGAAGGAAGCAGGATGT-3′
miR-545-3p	Forward: 5’-TGCGCTCAGCAAACATTTATTG-3’
	Reverse: 5’-CCAGTGCAGGGTCCGAGGTATT-3’
GAPDH	Forward: 5’-GGGAAACTGTGGCGTGAT-3’
	Reverse: 5’-GAGTGGGTGTCGCTGTTGA-3’
U6	Forward: 5’-CGCTTCGGCAGCACATATAC-3’
	Reverse: 5’-AAATATGGAACGCTTCACGA-3’

### Cell proliferation and invasion assays

Three thousand cells/well were seeded in a 96-well plate and cultured for the indicated times. The cell proliferation rate was measured after an additional 2 h of incubation with the Cell Counting kit-8 (CCK-8) reagent by taking the absorbance of cultured cells at 450 nm using a microplate reader [36].

For cell invasion analysis, HCC827, and NCI-H1650 cells (1 × 10^5^ cells/ml) were seeded into 48-well microchemotaxis Boyden chambers containing 12 μm pore membranes (Corning, UK) pre-treated with 10 μg/ml Matrigel and cultured for 48 h. The uninvaded cells were removed and the invaded cells were fixed with methanol followed by staining with 0.1% crystal violet for 20 min. The stained cells were counted and assessed in five randomly selected fields, and the data were obtained from triplicate experiments [37].

### RNA immunoprecipitation

HCC827 and NCI-H1650 cells were lysed and fractionated for immunoprecipitation of endogenous FAM83H-AS1 and miR-545-3p, respectively. The protein A-Sepharose beads were incubated with positive antibody Ago2, negative antibody IgG for 30 min at 4°C. The fractioned cell extracts were incubated with treated beads for 6 h at 4°C. The beads were then washed six times and further incubated with 0.1% sodium dodecyl sulfate (SDS)/0.5 mg/mL proteinase K for 30 min at 55°C to extract the RNA-protein complexes. The qPCR was used to evaluate FAM83H-AS1 and miR-545-3p enrichment [38].

### Luciferase report assay

The wide-type (WT) complementary sequence of FAM83H-AS1 or 3ʹUTR HS6ST2 to miR-545-3p and the matched mutant (MUT) sequence were amplified and ligated into pmirGLO vectors to produce FAM83H-AS1-WT, FAM83H-AS1-MUT, 3ʹUTR HS6ST2-WT and 3ʹUTR HS6ST2-MUT luciferase vectors. These constructed vectors were accompanied by miR-545-3p mimic or mimic NC to transfect HCC827 and NCI-H1650 cells. Finally, the relative luciferase activities were measured after 48 h of transfection using a Dual-Luciferase Assay Kit (Yeasen, China) [39].

### Fluorescent in situ hybridization (FISH) and immunofluorescence

The assay was performed using a FAM83H-AS1-specific probe (GenePharma, China). The cells were immobilized with 4% paraformaldehyde and incubated overnight with FAM83H-AS1 probe. The cells were then washed with 3% bovine serum albumin (BSA) and incubated with the HS6ST2 antibody (Cat#: ab122220, Abcam, USA). Next, the cells were incubated with Alexa Fluor 488 conjugated secondary antibodies (Invitrogen, USA) and counterstained using 4, 6-diamidino-2-phenylindole (DAPI). The final images were observed using a confocal microscope [40].

### Western blotting

HCC827 and NCI-H1650 cells were harvested and lysed in lysis buffer containing phenylmethylsulfonyl fluoride (PMSF) and protease inhibitors for 15 min on ice. The cell lysate was centrifuged at 12,000 × *g* and 4°C for 10 min. The proteins were quantified using a bicinchoninic acid protein assay kit (Merck, USA). Equal amounts of protein were loaded and electrophoresed on 10% SDS-polyacrylamide gel electrophoresis (PAGE) and then transferred onto a polyvinylidene fluoride (PVDF) membrane. After transfer, the membrane was blocked with 5% nonfat milk for 1 h at room temperature and then probed with anti- HS6ST2 (1:1000, Cat#: ab122220) and anti-GAPDH antibodies (1:1000; Cat#: ab8245) at 4°C overnight, and incubated with the HRP-conjugated secondary antibody for 1.5 h at room temperature. Protein bands were visualized using an ECL advance kit (Amersham, UK) [41].

### In vivo xenograft model

The *in vivo* experiments were approved by the Animal Research Ethics Committee of Tianjin Medical University Cancer Institute and Hospital (Tianjin, China). The 5-week-old female BALB/c nude mice were purchased from the Experimental Animal Center of Wuhan University (Wuhan, China) and kept under pathogen-free conditions in type IV Makrolon cages (six mice per cage) with an airflow cabinet at 23°C, 12 h/12 h day/night cycle. Sterilized food and acidified water were available at any time. HCC827 cells (2 × 10^7^ cells) were injected subcutaneously into the right flank of each mouse. Tumor size was calculated and recorded weekly. The tumors were extracted, photographed, and weighed after 6 weeks [42].

### Statistical analysis

GraphPad Prism software was used for the statistical analyses. The data were analyzed for statistical significance using the Student’s unpaired t-test. Statistical analysis between two or more groups was performed using two-way analysis of variance (ANOVA). Pearson’s tests were used to assess the correlation between miR-545-3p and FAM83H-AS1 or HS6ST2 expression in NSCLC tissues. Data were obtained in triplicate and presented as mean ± standard deviation (SD). Statistical significance was set at p < 0.05.

## Results

In present study, the effects of FAM83H-AS1, miR-545-3p, and HS6ST2 on proliferation, invasion, and tumor growth of NSCLC cells were investigated. FAM83H-AS1 and HS6ST2 are up regulated in NSCLC, whereas miR-545-3p is down regulated. Low FAM83H-AS1 expression inhibits the malignant behavior of NSCLC cells by sponging miR-545-3p. As a direct target of miR-545-3p, HS6ST2 can eliminate the malignant behavior of NSCLC cells dependent on miR-545-3p.

### FAM83H-AS1 regulates miR-545-3p/HS6ST2 axis in NSCLC

**Figure 1.**
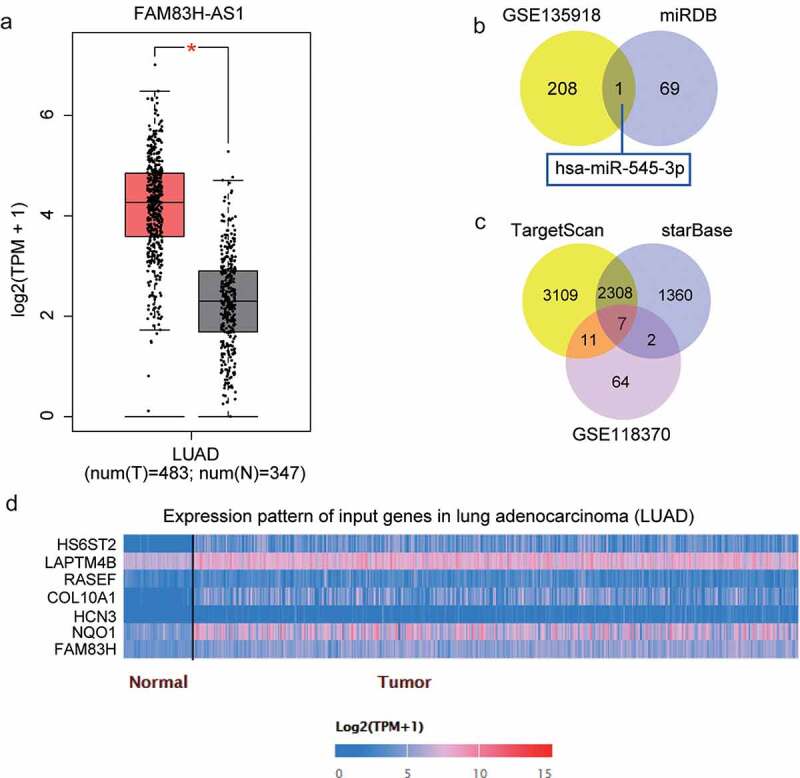
miR-545-3p/HS6ST2 axis might be the downstream of FAM83H-AS1 in NSCLC. (a) The overexpression of FAM83H-AS1 in LUAD samples based on GEPIA database. LUAD, lung adenocarcinoma. (b) miR-545-3p with downregulation in lung cancer was predicted to bind to FAM83H-AS1. GSE135918 was the miRNA expression profile to screen the downregulated miRNAs with adj.P < 0.05 and logFC<-2. miRDB was used to predict the miRNAs sponged by FAM83H-AS1. (c) Seven key genes were overlapped from TargetScan, starBase and GSE118370. TargetScan and starBase were used to predict the genes targeted by miR-545-3p. GSE118370 was the mRNA expression profile to screen the upregulated miRNAs with adj.P < 0.05 and logFC>2. (d) The expression of seven overlapped genes in lung adenocarcinoma samples based on the TCGA database.

According to the data gathered from the GEPIA database, FAM83H-AS1 is overexpressed in lung adenocarcinoma (Figure 1(a)) and its promotive effect on lung cancer has been previously reported [25,43]. However, the molecular mechanisms involving the downstream regulation have not been investigated. The miRDB prediction revealed that 70 miRNAs could bind to FAM83H-AS1. With adj.P < 0.05 and logFC<-2, 209 down regulated miRNAs were screened from GSE135918 that included lung cancer and non-tumor samples. The miR-545-3p expression was overlapped after Venny 2.1.0 analysis (Figure 1(b)). TargetScan and starBase were used to predict the target genes of miR-545-3p, and GSE118370 from GEO DataSets was used to screen the up regulated genes with adj. P < 0.05, logFC>2. Finally, the expression of seven genes (Figure 1(c)) was found to be overlapping in lung adenocarcinoma and normal samples (Figure 1(d)) from the TCGA database. Of these, HS6ST2, COL10A1, and NQO1 were significantly up regulated in tumor tissues. Adequate findings on COL10A1 and NQO1 in lung cancer have been reported [44–47]; therefore, HS6ST2 was identified as our gene of interest.

### FAM83H-AS1 inhibition suppressed lung cancer cell proliferation and invasion in vivo

**Figure 2.**
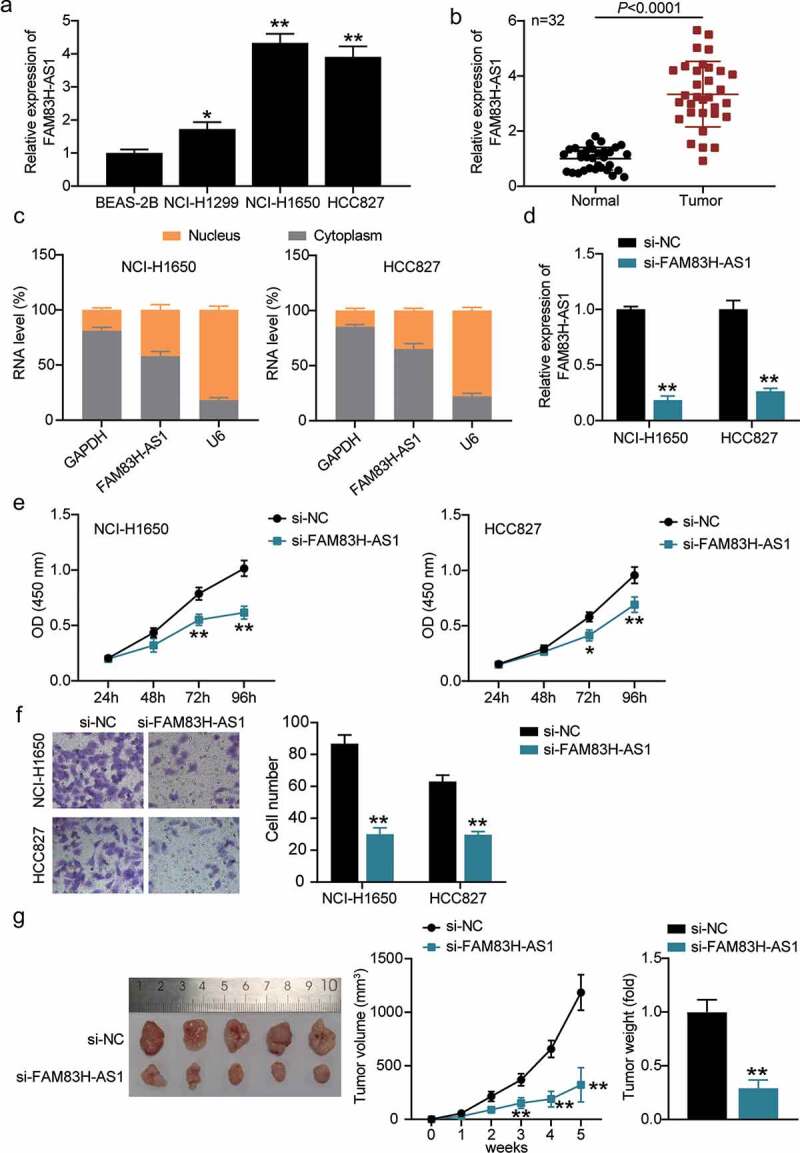
FAM83H-AS1 is vital for NSCLC pathogenesis. (a) The relative mRNA expression level of FAM83H-AS1 in normal lung cancer cells and NSCLC cell lines; (b) The expression levels of FAM83H-AS1 in 32 samples of normal lung tissues and NSCLC tissues. (c) Cytoplasmic and nuclear expression of FAM83H-AS1 in NSCLC cells. (d) The knockdown effect on FAM83H-AS1 relative gene expression in NSCLC cells. (e) The effect of FAM83H-AS1 knockdown on NSCLC cells’ proliferation. (f) The effect of FAM83H-AS1 knockdown on the NSCLC cells’ invasion. (g) The effect of FAM83H-AS1 knockdown on the tumor weight and volume in nude mice bearing the NSCLC HCC827 cells. Data are represented as mean ± SD. *P < 0.05, **P < 0.001, vs. si-NC.

The expression levels of FAM83H-AS1 in healthy and tumorous lung cells were compared. FAM83H-AS1 was up regulated in NCI-H1299, HCC827, and NCI-H1650 lung cancer cells compared with that in BEAS-2B cells (Figure 2(a)). We further analyzed the correlation between FAM83H-AS1 expression levels and clinico-pathological features (Supplementary Table 1). High expression of FAM83H-AS1 was strongly correlated with TNM stage but not with age, sex, tumor size, differentiation, lymph node metastasis, metastasis, histology type, and smoking. In addition, FAM83H-AS1 was highly expressed in tumor tissues than healthy tissues (Figure 2(b)). Considering its high expression in HCC827 and NCI-H1650 cells, we tested its localization in NSCLC cells. The results demonstrated that FAM83H-AS1 was more prominent in the cytoplasm of HCC827 and NCI-H1650 cells than in the nucleus (Figure 2(c)), suggesting its different role in the cytoplasm. We designed si-FAM83H-AS1 to knockdown FAM83H-AS1 resulting in significant inhibition of its expression in NSCLC cells compared to the negative control group (Figure 2(d)). We then observed the effect of silencing FAM83H-AS1 on cell proliferation and invasion and found invasive and proliferative defects in HCC827 and NCI-H1650 cells (Figure 2(e,f)). Additionally, si-FAM83H-AS1 reduced the tumor size in nude mice with HCC827 xenograft (Figure 2(g)). Collectively, FAM83H-AS1 possibly plays distinct regulatory roles in the pathogenesis of lung cancer.

### miR-545-3p is the target of FAM83H-AS1

**Figure 3.**
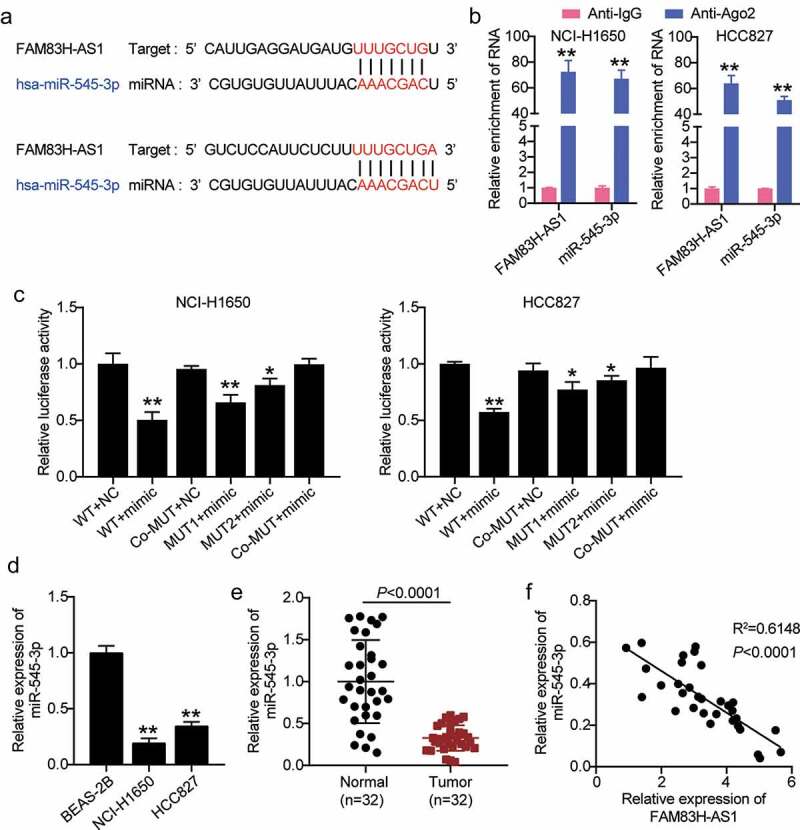
miR-545-3p is negatively correlated to FAM83H-AS1. (a) In silico prediction of FAM83H-AS1 target (b) Comparison of the different relative expression of FAM83H-AS1 and miR-545-3p in NSCLC cells by RNA RIP assays. (c) Luciferase activity of FAM83H-AS1-WT or FAM83H-AS1-MUTcontroling in NSCLC cells along with miR-545-3p mimic or mimic NC transfection. (d) The expression level of miR-545-3p in normal lung cells and NSCLC cells. (e) The expression level of miR-545-3p in 32 samples of normal lung tissues and NSCLC tissues. (f) A negative correlation was found between miR-545-3p and FAM83H-AS1 in NSCLC samples. Data are represented as mean ± SD. * *P* < 0.05,** *P* < 0.001.

Next, we used *in silico* analysis to predict potential targets of FAM83H-AS1. As shown in Figure 3(a), miR-545-3p is a potential target of FAM83H-AS1. The RNA RIP assays revealed elevated levels of FAM83H-AS1 and miR-545-3p in HCC827 and NCI-H1650 cells (Figure 3(b)). Luciferase analysis was performed to confirm the relationship between FAM83H-AS1 and miR-545-3p expression. The results showed that luciferase activity decreased after co-transfection of miR-545-3p mimic with FAM83H-AS1-WT, while it was unaffected after co-transfection of miR-545-3p mimic with FAM83H-AS1-MUT (Figure 3(c)). In contrast, high expression of miR-545-3p was observed in healthy lung cells and tissues than in tumor cells (Figure 3(d,e)). Simultaneously, FAM83H-AS1 expression was negatively correlated with the expression of miR-545-3p (Figure 3(f)).

### miR-545-3p inhibitor reverses the FAM83H-AS1 knockdown induced lung cancer suppression

**Figure 4.**
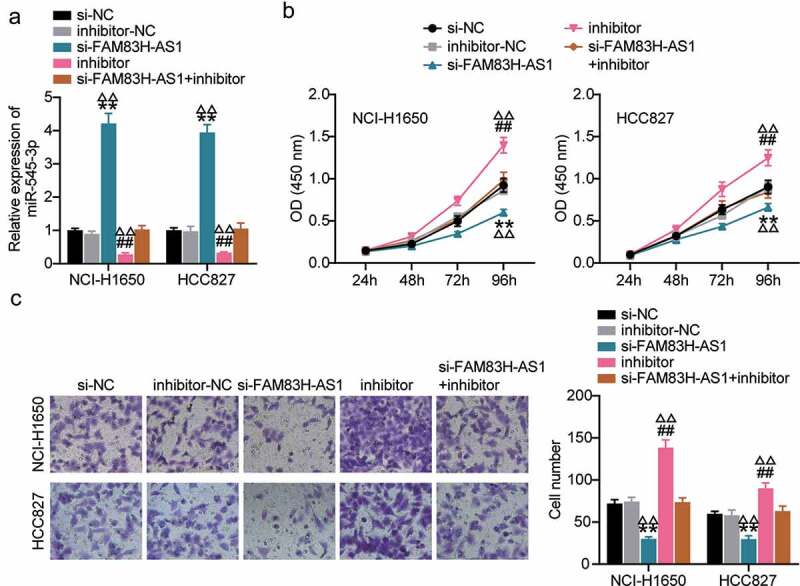
MiR-545-3p inhibitor reverses the suppression of FAM83H-AS1 knockdown on lung cancer cell progression. (a) The effect of FAM83H-AS1 knockdown on the miR-545-3p expression level. (b) CCK8 assay determined the effect of inhibiting the miR-545-3p and FAM83H-AS1 knockdown on the NSCLC cell proliferation. (c) The effect of inhibiting the miR-545-3p and FAM83H-AS1 knockdown on the NSCLC cell invasion. Data are represented as mean ± SD. *P < 0.05, **P < 0.001, vs.Si-NC; #*P* < 0.05, ##*P* < 0.001, vs. inhibitor-NC; Δ*P*<0.05, ΔΔ*P*<0.001, vs.si-FAM83S-AS1+ inhibitor.

We analyzed the competing endogenouse RNA (ceRNA) activity of FAM83H-AS1 against miR-545-3p in the progression of lung cancer *in vitro*. In HCC827 and NCI-H1650 cells, FAM83H-AS1 silencing relieved the inhibition of miR-545-3p expression caused by miR-545-3p inhibitor (Figure 4(a)). The outcome of CCK8 demonstrated that inhibiting miR-545-3p increased lung cancer cell proliferation, while the combination of si-lnc and miRNA inhibitor neutralized the inhibitory effect of miR-545-3p inhibitor (Figure 4(b)). To confirm these results, we evaluated the effects of miR-545-3p inhibition on cancer progression. Our results indicated that compared to the negative control group, miR-545-3p inhibition increased lung cancer cell invasion significantly, while additional transfection with si-FAM83H-AS1 significantly retracted the increased cell invasion by miR-545-3p inhibitor (Figure 4(c)). These results indicated a correlation between FAM83H-AS1 and miR-545-3p.

### miR-545-3p physically binds to HS6ST2 3ʹUTR

**Figure 5.**
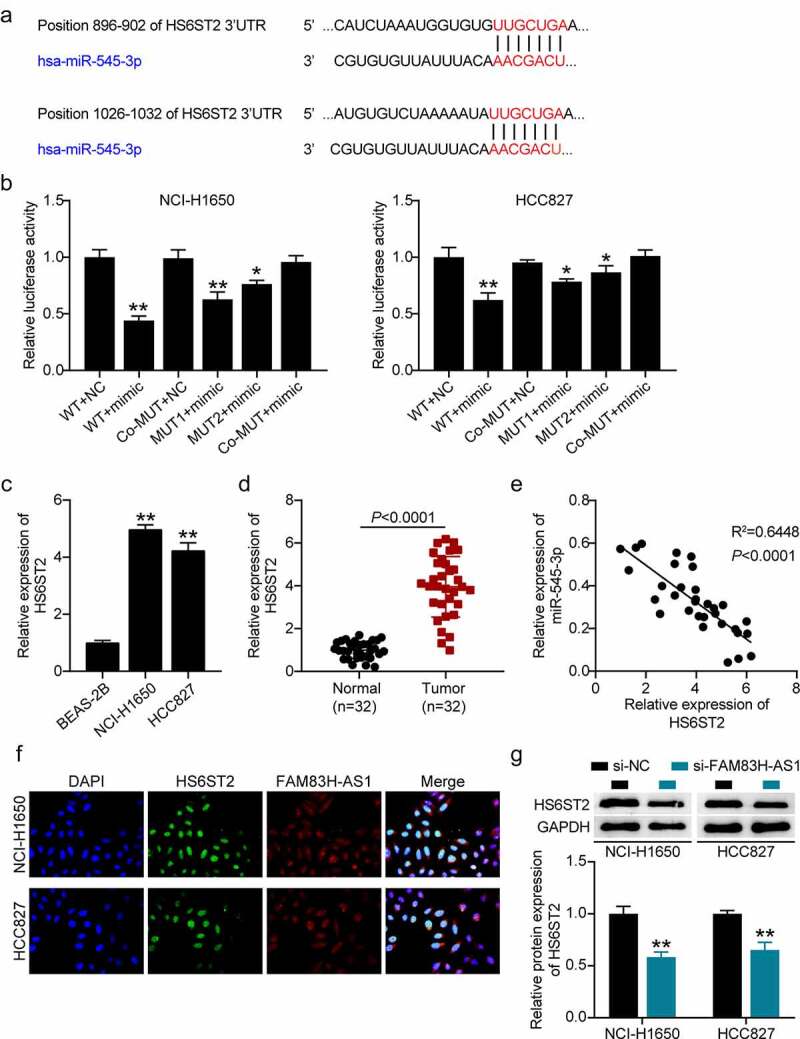
miR-545-3p is a target of HS6ST2. (a) Target scan prediction of the consequential pairing region of miR-545-3p. (b) Luciferase activity of HS6ST2 3ʹUTR-WT or HS6ST2-3ʹUTR-MUTcontroling in NSCLC cells along with miR-545-3p mimic or mimic NC transfection. (c) The expression level of HS6ST2 in 32 samples of normal lung tissues and NSCLC tissues. (d) The expression level of HS6ST2 in normal lung cells and NSCLC cells. (e) There was a negative correlation between the expression of HS6ST2 and miR-545-3p. Data are represented as mean ± SD. * *P* < 0.05, ** *P* < 0.001. (f) FISH assay displayed the co-location of FAM83H-AS1 and HS6ST2. (g) Western blotting detected the protein expression of HS6ST2 in NSCLC cells with the transfection of si-NC or si-FAM83H-AS1. ** *P* < 0.001, vs. Si-NC.

The potential target of miR-545-3p was identified using *in silico* analysis (Figure 5(a)) and target relationship between miR-545-3p and the 3’-UTR HS6ST2 was validated by luciferase reporter assays (Figure 5(b)). We found that HS6ST2 was highly expressed in the NSCLC tissues (Figure 5(c)), HCC827, and NCI-H1650 cell lines (Figure 5(d)). Interestingly, a negative correlation was observed between miR-545-3p and HS6ST2 expression (Figure 5(e)) suggesting that miR-545-3p may target HS6ST2. The FISH assay confirmed the co-localization of FAM83H-AS1 and HS6ST2 in HCC827 and NCI-H1650 cells (Figure 5(f)). Moreover, Western blotting demonstrated a decrease in HS6ST2 protein levels in HCC827 and NCI-H1650 cells after FAM83H-AS1 silencing (Figure 5(g)).

### The miR-545-3p inhibition promoted HS6ST2 expression and lung cancer progression

**Figure 6.**
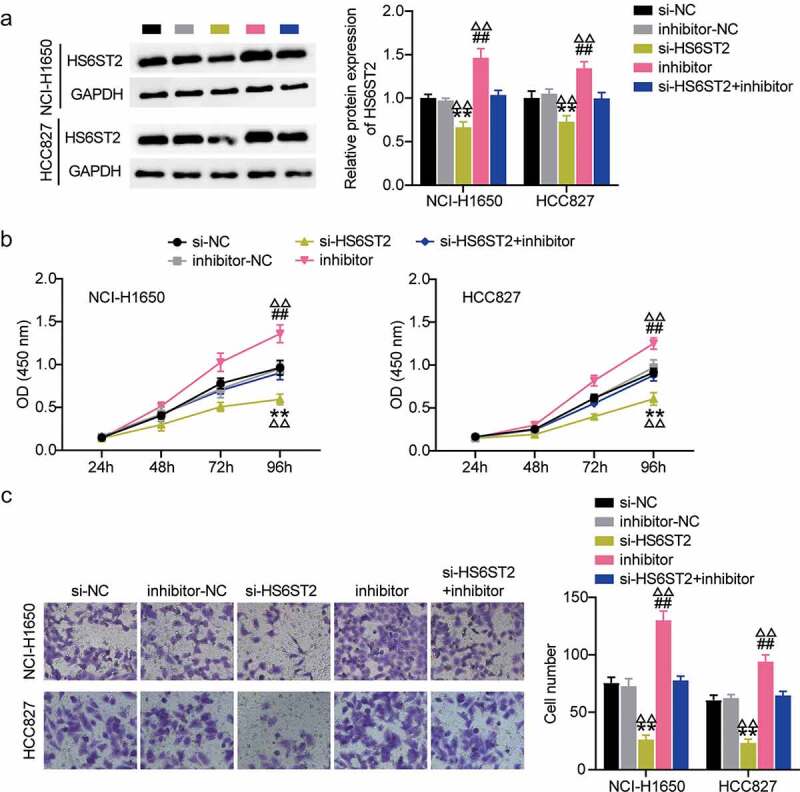
HS6ST2 is crucial for NSCLC cell survival. (a) Western blot analysis was utilized to determine the effect of miR-545-3p inhibition on HS6ST2 protein expression in NSCLC cells. (b) CCK8 assay was used to determine the effect of miR-545-3p inhibition and HS6ST2 knockdown on NSCLC cell proliferation. (c) The effect of inhibiting the miR-545-3p and HS6ST2 knockdown on the NSCLC cell invasion. Data are represented as mean ± SD. * *P* < 0.05, ** *P* < 0.001, vs.Si-NC; # *P* < 0.05, ##*P* < 0.001, vs. inhibitor-NC; Δ*P*<0.05, ΔΔ*P*<0.001, vs.si-HS6ST2+ inhibitor.

We further investigated the role of HS6ST2 in lung cancer progression. Western blot analysis showed that inhibition of miR-545-3p promoted HS6ST2 protein expression. Most importantly, it relieved the inhibitory effect of si-HS6ST2 on HS6ST2 expression levels (Figure 6(a)). These results highlight the crucial regulatory role of miR-545-3p on HS6ST2 expression. It also indicated that HS6ST2 silencing could suppress cell proliferation and invasions demonstrated by CCK8 and trans well invasion assays; however, this suppressive effect of HS6ST2 silencing was counteracted by simultaneous transfection with si-HS6ST2 and miR-545-3p inhibitor (Figure 6(b,c)).

## Discussion

Recent studies reported that lncRNAs are dysregulated in different cancer types leading to aberrant cell functions. They can act as tumor suppressors and oncogenes in various cancer types [48,49]. FAM83H-AS1 is one of the few dysregulated lncRNAs that are involved in the progression of lung cancer [43,50]. It is significantly overexpressed in tumorous lung cells than healthy cells and correlated with the worst lung patient survival [25]. Additionally, knockdown of FAM83H-AS1 decreases the cell migration, invasion, and proliferation [25]. Our experimental results demonstrated that FAM83H-AS1 knockdown inhibited cell proliferation in HCC827, NCI-H1650 cell lines, and reduced tumor size *in vivo*; thus validating the previous findings.

The expression of miRNAs is a distinctive feature of lung cancer. It can sustain proliferative signaling, evade growth suppressors, avoid immune destruction and tumors promoting inflammation, resist cell death mechanisms, deregulate cell energetics, activate invasion and metastasis, and induce angiogenesis [51]. Increased attention has been drawn toward miR-545-3p in human diseases and cancers. Down regulation has been reported to promote pancreatic cancer cell growth [52] and tumor progression in oral squamous cell carcinoma [53] and can also be used as a biomarker for the early diagnosis of Alzheimer’s disease [54]. miRNA-545-3p has been reported to induce cell cycle arrest and apoptosis in lung cancer by regulating CDK4 and cyclin D1 [55]. Besides, the expression of miR-545-3p was decreased in NSCLC tissues, and its overexpression suppressed the proliferation, invasion, and migration of NSCLC cells [56]. The results of our cell functional assays demonstrated that the miRNA-545-3p inhibitor promoted NSCLC cell proliferation and migration. In present study, we explored the interaction between miRNA-545-3p and FAM83H-AS1. First, luciferase reporter and RNA immunoprecipitation assays were performed to validate the predicted physical binding between miRNA-545 and FAM83H-AS1. Interestingly, we examined the down regulation of miRNA-545 and its negative correlation with FAM83H-AS1 in NSCLC tissues, which further supports the sponging between them. Not surprisingly, FAM83H-AS1 increased minimal miRNA-545-3p expression in HCC827 and NCI-H1650 cell lines treated with the miRNA-545-3p inhibitor. Cell functional assays revealed that FAM83H-AS1 silencing offset the proliferation and invasion of HCC827 and NCI-H1650 cell lines after reduction of endogenous miRNA-545-3p. Therefore, FAM83H-AS1 sponges miRNA-545-3p and suppresses its inhibitory effect on NSCLC cell proliferation and invasion.

Emerging evidence suggests that HS6ST2 is involved in biological functions of cancer cells [30,57–61]. Also, HS6ST2 has been found to be overexpressed in lung cancer and is identified as an inferior prognosticator [34]. However, HS6ST2 expression, regulation, and clinical significance in lung cancer have not yet been elucidated. In the present study, we found that HS6ST2 silencing reduced the proliferative and invasive phenotypes of NSCLC cells, suggesting its role in promoting lung cancer malignancy. Furthermore, HS6ST2 was confirmed as the target of miRNA-545-3p using luciferase reporter assays. Again, we found that the miRNA-545-3p inhibitor increased HS6ST2 expression, while co-silencing HS6ST2 and inhibiting miRNA-545-3p restored the suppression of cell proliferation and invasion caused by the miRNA-545-3p inhibitor.

Our results suggested that FAM83H-AS1 regulates NSCLC progression by regulating the HS6ST2/miRNA-545-3p axis. For the first time, we report HS6ST2 as a potential target of miRNA-545-3p and elucidate its role in lung cancer proliferation. The present study had some limitations. We investigated the role of FAM83H-AS1 in the HS6ST2/miRNA-545-3p axis *in vivo* and performed a detailed analysis of the underlying mechanism; however, we also need to validate our results regarding the different mechanisms of cell death. In conclusion, our data provide a novel target for miRNA-545-3p and HS6ST2 and elucidate the potential role of HS6ST2 in lung cancer proliferation. These results identify a novel target for the treatment of NSCLC and provide a better understanding of NSCLC progression.

## Conclusion

We highlighted the vital role of FAM83H-AS1 in NSCLC progression both *in vitro* and *in vivo*. Our results demonstrate that FAM83H-AS1 contributes to NSCLC progression via the miR-545-3p/HS6ST2 axis. These findings reveal a novel mechanism for the progression of NSCLC and provide a novel potential target for the treatment of lung cancer.

## Highlights

- FAM83H-AS1 is highly expressed in NSCLC
- Knockdown of FAM83H-AS1 inhibits cancer cell invasion and proliferation thereby reducing tumor growth
- FAM83H-AS1 targets miR-545-3p to regulate HS6ST2

## Supplementary Material

Supplemental Material

## Data Availability

The datasets used and/or analyzed during the current study are available from the corresponding author on reasonable request http://gepia.cancer-pku.cn/.
