# Supplementary material for: Role of lncRNA FAM83H antisense RNA1 (FAM83H-AS1) in the progression of non-small cell lung cancer by regulating the miR-545-3p/heparan sulfate 6-O-sulfotransferase (HS6ST2) axis
Source: Bioengineered. 2022 Mar 9;13(3):6476–89. doi: 10.1080/21655979.2022.2031668 (PMC8973780; doi:10.1080/21655979.2022.2031668)
Supplement: Supplemental Material [file KBIE_A_2031668_SM4833.zip › supplementary/Supplementary Table 1_revised.docx]

Supplementary Table 1. Correlation Between Clinicopathologic Features and FAM83H-AS1 high or low expression in NSCLC Patients.

| Clinicopathological parameters | N=32 | FAM83H-AS1 expression | | P-value |
| --- | --- | --- | --- | --- |
|  |  | High (N=16) | Low (N=16) |  |
| Age (years) |  |  |  | 0.458 |
| ≤65 | 21 | 9 | 12 |  |
| >65 | 11 | 7 | 4 |  |
| Gender |  |  |  | 0.704 |
| Male | 22 | 12 | 10 |  |
| Female | 10 | 4 | 6 |  |
| Tumor size |  |  |  | 0.273 |
| ≤3 cm | 12 | 4 | 8 |  |
| >3 cm | 20 | 12 | 8 |  |
| Differentiation |  |  |  | 0.685 |
| Well/moderate | 24 | 13 | 11 |  |
| Poor | 8 | 3 | 5 |  |
| Lymph node metastasis |  |  |  | 0.157 |
| N0 | 17 | 6 | 11 |  |
| N1-3 | 15 | 10 | 5 |  |
| Metastasis |  |  |  | 0.600 |
| M0 | 28 | 13 | 15 |  |
| M1 | 4 | 3 | 1 |  |
| TNM stages |  |  |  | 0.029 |
| I/II | 19 | 6 | 13 |  |
| III-IV | 13 | 10 | 3 |  |
| Histology type |  |  |  | 0.285 |
| Adenocarcinoma | 18 | 11 | 7 |  |
| Squamous carcinoma | 14 | 5 | 9 |  |
| Smoking |  |  |  | 0.273 |
| Yes | 20 | 8 | 12 |  |
| No | 12 | 8 | 4 |  |
