# Supplementary material for: Role of lncRNA FAM83H antisense RNA1 (FAM83H-AS1) in the progression of non-small cell lung cancer by regulating the miR-545-3p/heparan sulfate 6-O-sulfotransferase (HS6ST2) axis
Source: Bioengineered. 2022 Mar 9;13(3):6476–89. doi: 10.1080/21655979.2022.2031668 (PMC8973780; doi:10.1080/21655979.2022.2031668)
Supplement: Supplemental Material [file KBIE_A_2031668_SM4833.zip › supplementary/Supplementary Table 2_revised.docx]

Supplementary Table 2：Transfection sequences used in this study

| Characteristic | Sequence (5’-3’) |
| --- | --- |
| si-FAM83H-AS1 | CCTCTTGTTATTGACCCTT |
| si- HS6ST2 | CCUCUCUGUCAUGCCUGAATT |
| si-NC | UUCUCCGAACGUGUCACGUTT |
| si- HS6ST2 | CCUCUCUGUCAUGCCUGAATT |
| miR-545-3p mimic | UCAGCAAACAUUUAUUGUGUGC |
| mimic-NC | UGGGCGUAUAGACGUGUUACAC |
| miR-545-3p inhibitor | GCACACAAUAAAUGUUUGCUGA |
| Inhibitor-NC | UUCUCCGAACGUGUCACGUdTdT |
